# Effect of physical therapy on the flexibility of the infrapatellar fat pad: A single-blind randomised controlled trial

**DOI:** 10.1371/journal.pone.0265333

**Published:** 2022-03-17

**Authors:** Takashi Kitagawa, Natsumi Ozaki, Yuma Aoki

**Affiliations:** Department of Physical Therapy, School of Health Sciences, Shinshu University, Matsumoto, Japan; Prince Sattam Bin Abdulaziz University, College of Applied Medical Sciences, SAUDI ARABIA

## Abstract

The infrapatellar fat pad plays a biomechanical role in the knee joint. After knee injury or surgery, its dynamics decrease because of an inflammatory response. Physical therapy might be one of the valuable treatments for the recovery of knee joint mobility. This study aimed to evaluate the immediate effect of physical therapy on the dynamics of the infrapatellar fat pad in healthy participants using ultrasonography. In this prospective, single-blind, randomised controlled trial, 64 healthy young participants were enrolled and randomly assigned to one of the following three interventions: manual therapy, hot pack treatment, and control. Ultrasound images of the infrapatellar fat pad were obtained before and after the intervention. The thickness change ratio of the infrapatellar fat pad was calculated to compare the changes between and within groups before and after the intervention. No significant inter-group differences were observed. The effect sizes were relatively small. Manual therapy or hot pack intervention might not have an immediate effect on infrapatellar fat pad flexibility in healthy participants. Thus, it is necessary to consider more intensive treatments to change the dynamics of the infrapatellar fat pad.

## Introduction

The infrapatellar fat pad (IPFP) is one of the four fat pads around the knee joint [1]. The IPFP is an intracapsular extra synovial structure [2]. Its role is thought to be biomechanical; it causes flexible deformation in knee joint motion, distributes pressure across the patellofemoral joint, and supports patellar stability [3, 4]. In patients with knee joint diseases, such as anterior cruciate ligament injury, the IPFP shows reduced flexibility [5]. Decreased flexibility of the IPFP is also associated with clinical symptoms, such as limited range of motion and anterior pain in the knee joint [6, 7]. Therefore, it is clinically important to maintain normal flexibility of the IPFP or recover the movement once it is reduced.

Several randomised controlled trials and a few meta-analyses have investigated therapeutic interventions for the IPFP [8]. However, most of these have focused on surgical rather than on conservative treatment. Numerous reports have compared the clinical outcomes of total knee arthroplasty with and without resection of the IPFP [9]. In terms of non-surgical treatment, other interventional studies have reported that the IPFP is a source of stem cells in stem cell therapy [10]. In clinical practice, the meagre presence of pain symptoms caused primarily by the IPFP is not an indication for surgical treatment such as IPFP resection. Conservative therapy is generally the first choice of treatment for these patients. Physical therapy, taping, strength training, gait training, injections, and manual therapy have been reportedly useful in conservative treatment for such patients [11, 12]. However, these interventions are often dependent on expert opinion.

To date, there have been few reports on conservative clinical treatments for improving flexibility and lesions of the IPFP, which are associated with anterior knee pain [7]. Conservative treatment, such as physical therapy, is an option for anterior knee pain [13, 14]. However, no studies have so far examined the effects of physical therapy intervention on the IPFP. Thus, this study aimed to evaluate the immediate effects of physical therapy on the dynamics of the IPFP in healthy participants using ultrasonography (US).

## Materials and methods

### Study design

This study was a single-blind, single-centre, pre-post evaluation, parallel randomised controlled trial. Sixty-five participants enrolled were healthy students from the university’s medical school recruited between September 2020 and December 2020 through on-campus announcement boards. A block randomisation method was used to assign all eligible participants to one of three groups by the evaluator: manual therapy group, hot pack group, or control group (**Table 1**). The exclusion criteria were as follows: (1) sensory disorders in their lower limbs; (2) a history of neurological or orthopaedic diseases of the spine and/or lower limbs; and (3) restricted knee joint extension or hypermobility for some reason. The intervener and evaluator operated independently. Another investigator was assigned to analyse the data. A prospective, randomised, open-blinded end-point study protocol was adopted for controlling information bias [15]. In other words, the data analyst was not informed about the groups to which the participants were allocated. All participants provided their informed consent. The study conformed to the Declarations of Helsinki and was approved by the institution’s medical research ethics committee. Our research protocol was registered on UMIN-CTR (trial number: UMIN000040075). Our data collection started on October 1 and ended on November 5, 2020.

**Table 1:** Characteristics of the participants.

Characteristic	Mean ± SD
**Age (years)**	20.7 ± 1.3
**Height (cm)**	165.1 ± 8.3*
**Weight (kg)**	56.2 ± 8.2
**BMI (kg/m** ^**2**^ **)**	20.6 ± 2.0*
**Thickness of the superficial part of the IPFP at 90° knee flexion (mm)**	8.8 ± 3.5*
**Thickness of the superficial part of the IPFP at 0° knee flexion (mm)**	4.4 ± 1.8*
**IPFP thickness change ratio (%)**	209.6 ± 70.1

*: The data is normally distributed.

SD, standard deviation; BMI, body mass index; IPFP, infrapatellar fat pad.

### Interventions

#### Manual therapy group

The participant was placed in a supine position and asked to relax both lower limbs. The examiner alternately pressed the IPFP of the participant from the lateral side to the medial side with as much pressure as possible and with a steady rhythm. The strength of the pressure toward the IPFP was such that the dynamics of the IPFP could be palpated with the other side finger. The examiner performed manual therapy for 3 min, considering the practical intervention in a clinical setting.

#### Hot pack group

The examiner performed the treatment using a hot pack (PE-26N, Yaesu Corporation, Takamatsu, Japan). The intervention time was set at 10 min, considering the practical intervention in clinical settings. The skin temperature was measured and monitored using a dermatometer before and after the intervention to confirm safety and the heat effect.

#### Control group

The participants were asked to relax their limbs for 10 min.

### Measurements and procedures

The weight, height, and body mass index were obtained at baseline. US data were obtained at baseline and after the interventions.

Measurements were made only on the right knee of the participants. US was performed with the participants placed in the sitting position, using a 5–18-MHz linear transducer (ARIETTA Prologue, Hitachi Aloka Medical, Tokyo, Japan). The thickness of the superficial part of the IPFP was measured at 90° and 0° knee flexion. Participants extended their knees from 90° to 0° actively. US of the IPFP was performed by an evaluator using a previously established measurement method [5]. ImageJ (National Institutes of Health, USA) was used for image analysis. The same evaluator measured the thickness of the superficial part of the IPFP at 90° and 0° knee flexion. Considering the dynamics of the IPFP during knee movement as the primary outcome, the ratio of the change in IPFP thickness between the two flexion angles was calculated using the following formula:

$$\text{IPFP}\;\text{thickness}\;\text{change}\;\text{ratio}=\frac{\text{thickness}\;\text{of}\;\text{the}\;\text{superficial}\;\text{part}\;\text{of}\;\text{the}\;\text{IPFP}\;\text{at}\;90{}^{\circ}\;\text{knee}\;\text{flexion}}{\text{thickness}\;\text{of}\;\text{the}\;\text{superficial}\;\text{part}\;\text{of}\;\text{the}\;\text{IPFP}\;\text{at}\;0{}^{\circ}\;\text{knee}\;\text{flexion}}$$

To confirm the reproducibility of the examinations, measurements were taken on eight knees with an interval of 1 week. The intra-examiner reliability (1,2) was 0.999 (0.993–1.000) at 90° of flexion and 0.962 (0.617–0.997) at 0° of flexion.

### Statistical analyses

Intention-to-treat analyses were conducted using IBM SPSS Statistics for Macintosh, ver. 27 (IBM Corp., Armonk, NY, USA). For the evaluation of variables, we planned to perform a two-way analysis of variance for dependent variables with normal distribution to investigate the interaction between the intervention and time and multiple comparison method for those without normal distribution. However, after checking the normality of distribution of our data, we found that the variables did not follow a normal distribution; thus, Wilcoxon signed-rank tests were used to compare the differences between the control group and manual therapy/hot pack groups after the intervention using the Dunnett method. Wilcoxon signed-rank tests were also used to compare the changes in the manual therapy and hot pack groups before and after the intervention using the Bonferroni method. Cohen’s d effect sizes were computed for all significant effects. The sample size was calculated using G*Power 3.1.9.7 (Heinrich Heine University, Dusseldorf). Under the assumption of using the two-way analysis of variance, the sample size for each group was calculated to require a total of 52 participants, assuming an effect size of 0.4, a significance level of <5%, and a power of 0.8. Assuming that some participants would declare that they cannot give consent for randomisation or would satisfy the exclusion criteria, a target of enrolling 65 participants was set. All statistical analyses were performed using IBM SPSS Statistics for Macintosh, ver. 26 (IBM Corp., Armonk, NY, USA).

### Patient and public involvement in research

The study participants were not involved in the design, conduct, interpretation, or translation of the current research.

## Results

**Fig 1:**
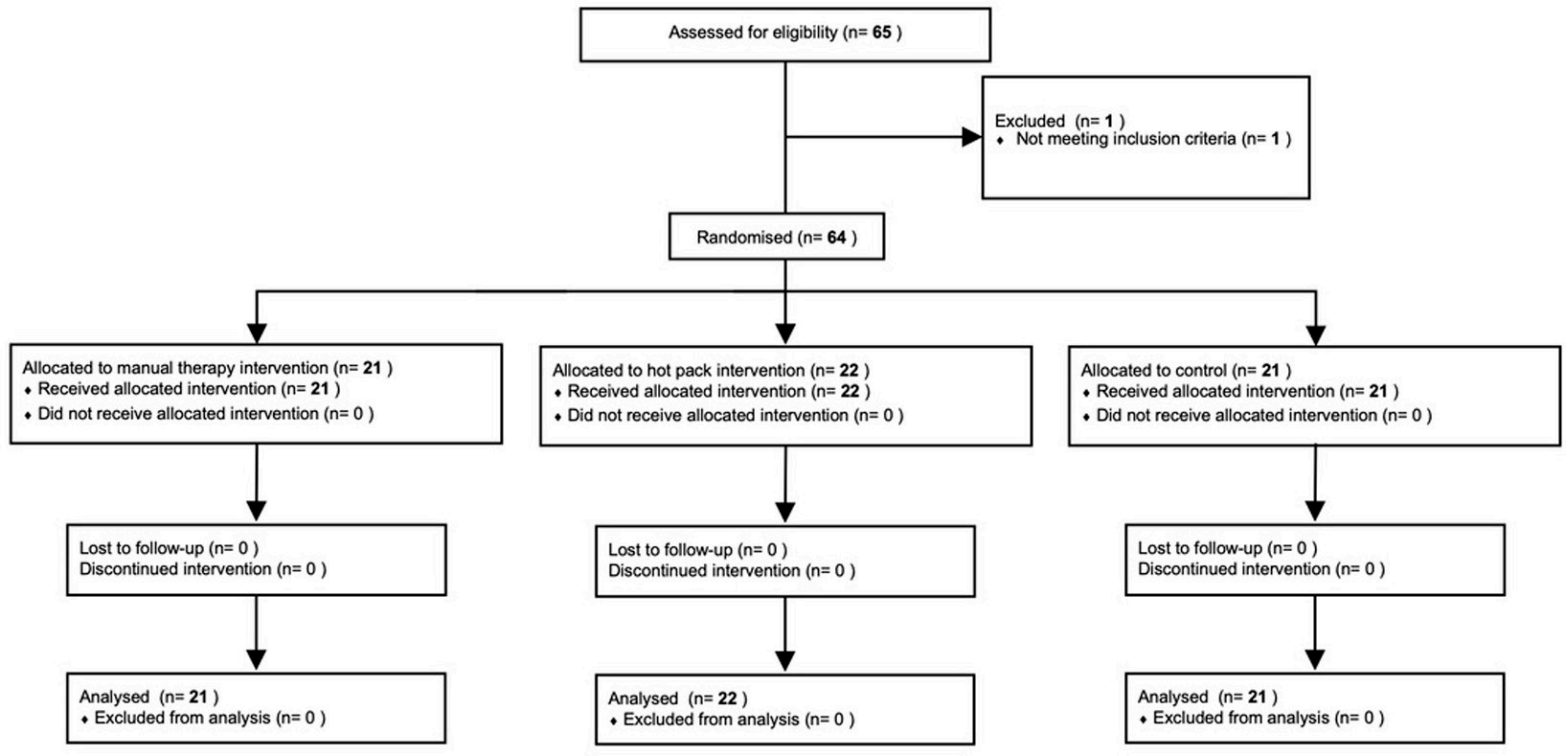
Participants’ progress throughout the trial.

There were no dropouts during the process from allocation to post-intervention (**Fig 1**).

**Fig 2:**
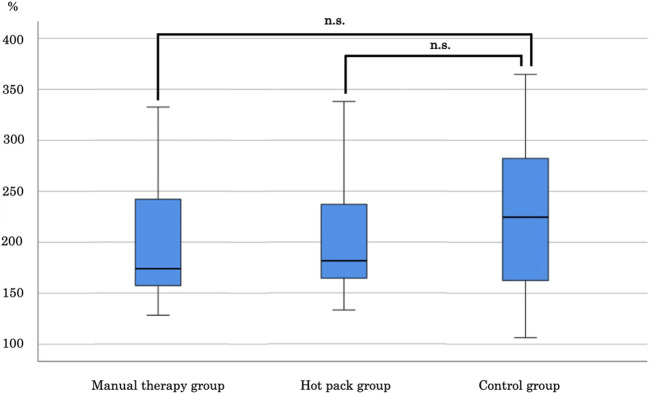
Comparison of the data between the manual therapy, hot pack, and control groups after the intervention. IPFP, infrapatellar fat pad; n.s., not significant.

Since the dependent variable was non-normally distributed, comparisons between the groups were made using the multiple comparison method. Bonferroni test was performed to determine the treatment effect after the intervention. No significant difference was found between the manual therapy and hot pack groups and the control group (**Fig 2**).

**Fig 3:**
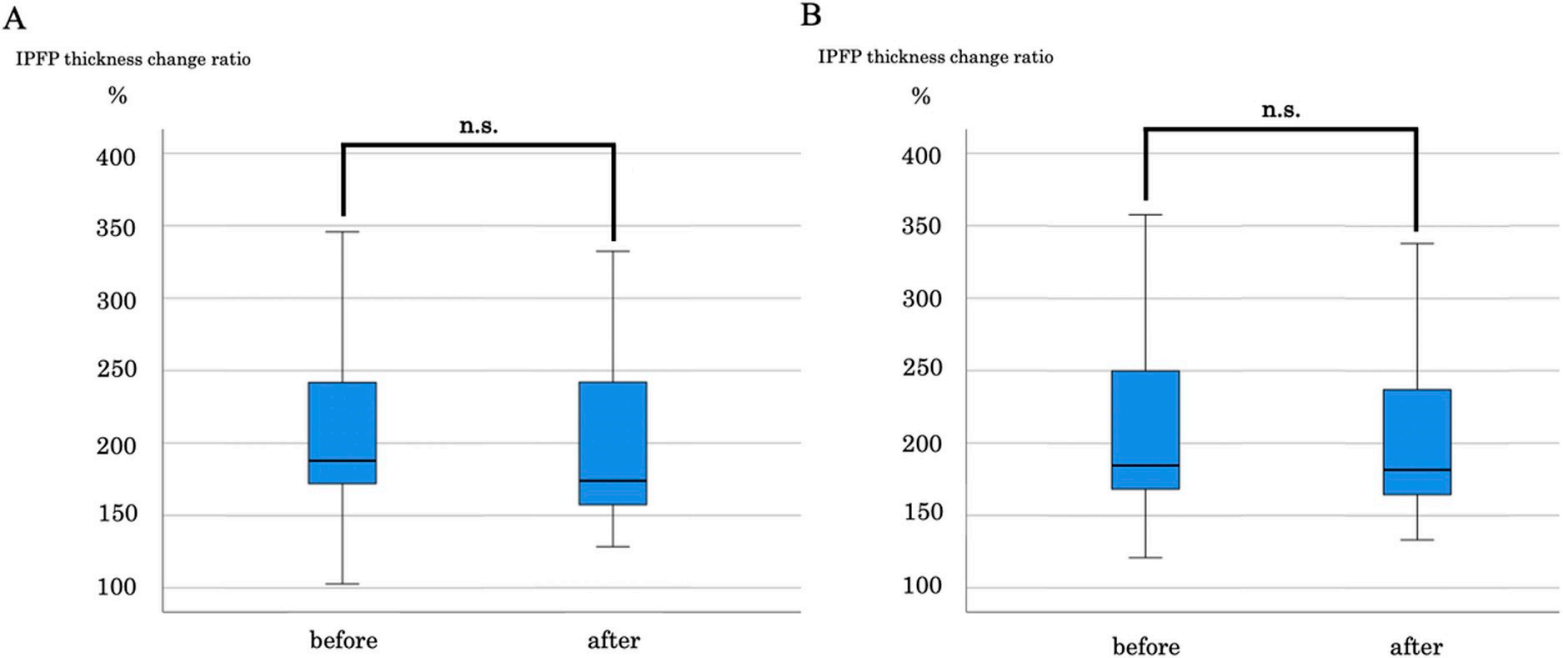
Respective changes before and after the two interventions. (A) Comparison of pre-and post-intervention IPFP thickness change ratio in the manual therapy group. (B) Comparison of pre-and post-intervention IPFP thickness change ratio in the hot pack group. IPFP, infrapatellar fat pad.

The effect sizes were -0.17 between the manual therapy and control groups and -0.14 between the hot pack and control groups. Dunnett test was performed to examine the change in the ratio before and after the manual therapy and hot pack interventions. The median values were compared before and after each intervention, and no significant difference was observed (**Fig 3**). The effect sizes were -0.02 and 0.16 in the manual therapy and hot pack groups, respectively.

## Discussion

In this study, we observed and verified the effects of massage and heat therapy on the dynamics of the IPFP, as performed in clinical practice. The type and intensity of the interventions used in this study had a little immediate effect on the dynamics of the IPFP. Although a small amount of change was observed, most of the effect sizes were extremely small, and the clinical significance and effect were negligible.

Previous studies have reported the usefulness of physical therapy, taping, muscle training, gait training, and injections as conservative treatments for IPFP disorders. In cases refractory to these treatments, operative treatments have been considered and performed [16, 17]. In this study, we focused on physical therapy among these treatment methods. To accumulate primary data, we conducted a randomised controlled trial of the effects of physiotherapy on the IPFP in healthy participants. Although there have been previous reports on physical therapy for IPFP lesions [11, 18], the objective effects of these interventions have not yet been examined. Results from a basic experiment in a rat model of knee joint contracture revealed that passive exercise might suppress fibrosis of the adipocytes in the IPFP [19]. In the manual therapy group of this study, we attempted to intervene with massage for a relatively short period considering the time required to provide physical therapy in actual clinical practice. However, the amount of change pre-and post-intervention and the difference in the amount of change between the massage therapy and control groups were negligible with probability of little clinical significance. Studies targeting muscles have reported improved tissue flexibility because of the thermal effects of hot packs [20]. In this study, we focused on the dynamic changes of the IPFP using US and observed whether the flexibility of the IPFP was changed by the thermal effect. The amount of change before and after the treatment and the difference in the amount of change between the hot pack and control groups were still very small and had little clinical significance. Regarding the intensity and time of therapeutic interventions, it is desirable to demonstrate that they are safe and to confirm that they are useful for patients [21]. The present study, which validates non-invasive interventions for the IPFP, is highly significant in terms of providing basic data as a phase I study. Additionally, the calculation of the effect size, rather than simply confirming the significant differences between the groups of interest-based on sample size calculations, as in conventional studies, is highly informative for similar studies in the future.

This study has a few limitations. First, although the data analysts were blinded, blinding the patients and therapists was not possible. Although various types of blinding are difficult in non-pharmacological studies, we should have devised a method of blinding for the investigators, such as hiding the changes on the skin surface due to hyperthermia [22]. Second, the short-term effects were only examined, so the medium- to long-term effects are unknown. However, since there was no significant short-term effect on the current intensity and volume, it may be necessary to reconsider the intensity and time of the intervention before extending the follow-up period in the future. While referring to previous long-term intervention studies with IPFP volume change as the outcome, intervention studies with a long-term duration that also included exercise and diet therapy should be considered [23]. Third, the study enrolled healthy participants. Since fibrosis of adipose tissue has been reported to be observed in the IPFP after patellar tendinopathy or anterior cruciate ligament reconstruction, there is a possibility that greater changes will be observed in patients [24]. We should also consider that the volume of the IPFP might change in patients as compared with that in healthy individuals [25]. Further studies are needed to confirm whether US is as good as magnetic resonance imaging (MRI) in measuring changes in IPFP thickness. Moreover, it is desirable to compare US findings with those of other imaging techniques, such as sagittal MRI. To improve the quality of the study, future studies may reconsider the interventions and consider blinding the evaluators to the patients.

## Conclusion

Manual therapy and hot packs demonstrated almost no immediate effect on IPFP flexibility changes in healthy participants. It is necessary to consider more intensive treatments to change the dynamics of the IPFP.

## Supporting information

S1 Checklist (DOCX)

S1 Data (XLS)

S1 File (DOCX)

S2 File (DOCX)

## References

[1] Clockaerts S, Bastiaansen-Jenniskens YM, Runhaar J, Van Osch GJ, Van Offel JF, Verhaar JA, et al. The infrapatellar fat pad should be considered as an active osteoarthritic joint tissue: A narrative review. Osteoarthr Cartil. 2010;18: 876–882. doi: 10.1016/j.joca.2010.03.014 20417297

[2] Jacobson JA, Lenchik L, Ruhoy MK, Schweitzer ME, Resnick D. MR imaging of the infrapatellar fat pad of Hoffa. RadioGraphics. 1997;17: 675–691. doi: 10.1148/radiographics.17.3.9153705 9153705

[3] Bohnsack M, Hurschler C, Demirtas T, Rühmann O, Stukenborg-Colsman C, Wirth CJ. Infrapatellar fat pad pressure and volume changes of the anterior compartment during knee motion: Possible clinical consequences to the anterior knee pain syndrome. Knee Surg Sports Traumatol Arthrosc. 2005;13: 135–141. doi: 10.1007/s00167-004-0561-1 15756618

[4] Mace J, Bhatti W, Anand S. Infrapatellar fat pad syndrome: A review of anatomy, function, treatment and dynamics. Acta Orthop Belg. 2016;82: 94–101. 26984660

[5] Kitagawa T, Nakase J, Takata Y, Shimozaki K, Asai K, Tsuchiya H. Use of ultrasonography to evaluate the dynamics of the infrapatellar fat pad after anterior cruciate ligament reconstruction: a feasibility study. J Med Ultrason (2001). 2019;46: 147–151. doi: 10.1007/s10396-018-0917-7 30456484

[6] Kitagawa T, Nakase J, Takata Y, Shimozaki K, Asai K, Toyooka K, et al. Relationship between the deep flexion of the knee joint and the dynamics of the infrapatellar fat pad after anterior cruciate ligament reconstruction via ultrasonography. J Phys Ther Sci. 2019;31: 569–572. doi: 10.1589/jpts.31.569 31417223 PMC6642903

[7] Kitagawa T, Nakase J, Takata Y, Shimozaki K, Asai K, Yoshimizu R, et al. Flexibility of infrapatellar fat pad affecting anterior knee pain 6 months after anterior cruciate ligament reconstruction with hamstring autograft. Sci Rep.: 21347. Sci Rep. 2020;10: 21347. doi: 10.1038/s41598-020-78406-y 33288779 PMC7721795

[8] Duan G, Liu C, Lin W, Shao J, Fu K, Niu Y, et al. Different factors conduct anterior knee pain following primary total knee arthroplasty: A systematic review and meta-analysis. J Arthroplasty. 2018;33: 1962-1971.e3. doi: 10.1016/j.arth.2017.12.024 29398258

[9] Sun C, Zhang X, Lee WG, Tu Y, Li H, Cai X, et al. Infrapatellar fat pad resection or preservation during total knee arthroplasty: A meta-analysis of randomized controlled trials. J Orthop Surg Res. 2020;15: 297. doi: 10.1186/s13018-020-01823-2 32758250 PMC7409474

[10] Dubey NK, Mishra VK, Dubey R, Syed-Abdul S, Wang JR, Wang PD, et al. Combating osteoarthritis through stem cell therapies by rejuvenating cartilage: A review. Stem Cells Int. 2018;2018: 5421019. doi: 10.1155/2018/5421019 29765416 PMC5885495

[11] Dragoo JL, Johnson C, McConnell J. Evaluation and treatment of disorders of the infrapatellar fat pad. Sports Med. 2012;42: 51–67. doi: 10.2165/11595680-000000000-00000 22149697

[12] Hannon J, Bardenett S, Singleton S, Garrison JC. Evaluation, treatment, and rehabilitation implications of the infrapatellar fat pad. Sports Health. 2016;8: 167–171. doi: 10.1177/1941738115611413 26502189 PMC4789926

[13] Bosshardt L, Ray T, Sherman S. Non-operative management of anterior knee pain: Patient education. Curr Rev Musculoskelet Med. 2021;14: 76–81. doi: 10.1007/s12178-020-09682-4 33523412 PMC7848041

[14] McClinton SM, Cobian DG, Heiderscheit BC. Physical therapist management of anterior knee pain. Curr Rev Musculoskelet Med. 2020;13: 776–787. doi: 10.1007/s12178-020-09678-0 33128200 PMC7661565

[15] Hansson L, Hedner T, Dahlöf B. Prospective randomized open blinded end-point (PROBE) study. A novel design for intervention trials. Blood Press. 1992;1: 113–119. doi: 10.3109/08037059209077502 1366259

[16] Doner GP, Noyes FR. Arthroscopic resection of fat pad lesions and infrapatellar contractures. Arthrosc Tech. 2014;3: e413–e416. doi: 10.1016/j.eats.2014.04.002 25126514 PMC4129979

[17] Kumar D, Alvand A, Beacon JP. Impingement of infrapatellar fat pad (Hoffa’s disease): Results of high-portal arthroscopic resection. Arthroscopy. 2007;23: 1180-1186.e1. doi: 10.1016/j.arthro.2007.05.013 17986405

[18] Doucette SA, Child DD. The effect of open and closed chain exercise and knee joint position on patellar tracking in lateral patellar compression syndrome. J Orthop Sports Phys Ther. 1996;23: 104–110. doi: 10.2519/jospt.1996.23.2.104 8808512

[19] Takeda K, Takeshima E, Kojima S, Watanabe M, Matsuzaki T, Hoso M. Daily and short-term application of joint movement for the prevention of infrapatellar fat pad atrophy due to immobilization. J Phys Ther Sci. 2019;31: 873–877. doi: 10.1589/jpts.31.873 31871369 PMC6879406

[20] Funk D, Swank AM, Adams KJ, Treolo D. Efficacy of moist heat pack application over static stretching on hamstring flexibility. J Strength Cond Res. 2001;15: 123–126. 11708695

[21] Lyauk YK, Jonker DM, Lund TM. Dose finding in the clinical development of 60 US Food and Drug Administration–approved drugs compared with learning vs. confirming recommendations. Clin Transl Sci. 2019;12: 481–489. doi: 10.1111/cts.12641 31254374 PMC6742935

[22] Armijo-Olivo S, Fuentes J, da Costa BR, Saltaji H, Ha C, Cummings GG. Blinding in physical therapy trials and its association with treatment effects: A meta-epidemiological study. Am J Phys Med Rehabil. 2017;96: 34–44. doi: 10.1097/PHM.0000000000000521 27149591

[23] Pogacnik Murillo AL, Eckstein F, Wirth W, Beavers D, Loeser RF, Nicklas BJ, et al. Impact of diet and/or exercise intervention on infrapatellar fat pad morphology: Secondary analysis from the Intensive Diet and Exercise for Arthritis (IDEA) Trial. Cells Tissues Organs. 2017;203: 258–266. doi: 10.1159/000449407 28222422 PMC5388564

[24] Murakami S, Muneta T, Ezura Y, Furuya K, Yamamoto H. Quantitative analysis of synovial fibrosis in the infrapatellar fat pad before and after anterior cruciate ligament reconstruction. Am J Sports Med. 1997;25: 29–34. doi: 10.1177/036354659702500106 9006688

[25] van der Heijden RA, de Vries BA, Poot DHJ, van Middelkoop M, Bierma-Zeinstra SMA, Krestin GP, et al. Quantitative volume and dynamic contrast-enhanced MRI derived perfusion of the infrapatellar fat pad in patellofemoral pain. Quant Imaging Med Surg. 2021;11: 133–142. doi: 10.21037/qims-20-441 33392017 PMC7719925

