# Supplementary material for: Effect of physical therapy on the flexibility of the infrapatellar fat pad: A single-blind randomised controlled trial
Source: PLoS One. 2022 Mar 17;17(3):e0265333. doi: 10.1371/journal.pone.0265333 (PMC8929552; doi:10.1371/journal.pone.0265333)
Supplement: S1 File — (DOCX) [file pone.0265333.s003.docx]

**Independent clinical research**

**Development of the evaluation of gliding properties of tissues around the knee joint and treatment methods using ultrasound image**

**Research plan**

Principal Investigator:

Takashi Kitagawa

Assistant Professor

Department of Physical Therapy, School of Health Sciences, Shinshu University

| Number of editions | Creation date |
| --- | --- |
| Version 1.0 | 30 January 2020 |
|  |  |
|  |  |
|  |  |
|  |  |

Table of Contents

[0. overview 5](#_Toc521433322)

[0.1. Schema 5](#_Toc521433323)

[0.2. Purpose and significance 5](#_Toc521433324)

[0.3. Object 5](#_Toc521433325)

[0.4. Target number of subjects enrolled and duration of study 5](#_Toc521433326)

[0.5. Research design 6](#_Toc521433327)

[0.6. Evaluation items 6](#_Toc521433328)

[0.7. Contact details 6](#_Toc521433329)

[1. purpose and significance 7](#_Toc521433330)

[2. background and rationale 7](#_Toc521433331)

[3. overview of the test drug 8](#_Toc521433332)

[4. eligibility criteria 8](#_Toc521433333)

[4.1. selection criteria 8](#_Toc521433334)

[4.2 Exclusion criteria 8](#_Toc521433335)

[4.3 Subjects for whom consent is required from a representative and the reasons for this 8](#_Toc521433335)

[5. research methods 9](#_Toc521433337)

[5.1 Research design 9](#_Toc521433338)

[5.2 Outline of the study 9](#_Toc521433339)

[5.3 How the intervention is implemented 9](#_Toc521433340)

[5.4 Provisions for concomitant medicines (combination therapy) 10](#_Toc521433341)

[5.5 What to do with subjects after completion of the study 10](#_Toc521433346)

[6. method of registration and allocation of subjects 10](#_Toc521433347)

[6.1 Registration of subjects 10](#_Toc521433348)

[6.2 Allocation methods and allocation adjustment factors 10](#_Toc521433349)

[7. endpoints 10](#_Toc521433350)

[7.1 Primary endpoints 10](#_Toc521433351)

[7.2 Secondary endpoints 11](#_Toc521433352)

[8. observation and examination items 11](#_Toc521433353)

[9. handling of adverse events 11](#_Toc521433354)

[10. target number of people to be registered 11](#_Toc521433361)

[11. statistical matters 11](#_Toc521433362)

[11.1 Basis for setting the target number of people to be enrolled 11](#_Toc521433363)

[11.2 Statistical analysis methods 12](#_Toc521433364)

[11.3 Analysis items and methods 12](#_Toc521433365)

[11.3.1 Overview of the analysis 12](#_Toc521433366)

[11.3.2 Hypothesis-testing analysis of primary endpoints 12](#_Toc521433367)

[11.3.3 Analysis of secondary endpoints 12](#_Toc521433368)

[11.3.4 Hypothesis exploratory analysis of primary and secondary endpoints 13](#_Toc521433369)

[12. completion and submission of the subject report form 13](#_Toc521433370)

[13. monitoring 13.](#_Toc521433373)

[14. audit 13.](#_Toc521433374)

[15. ethical matters 13](#_Toc521433375)

[15.1 Rules to be observed 13](#_Toc521433376)

[15.2 Informed consent 13](#_Toc521433377)

[15.3 Protection of personal data 14](#_Toc521433378)

[15.4 Preservation of samples and information, etc. 15](#_Toc521433379)

[16. record-keeping matters relating to the provision of samples and information 15](#_Toc521433381)

[17. changes to the research protocol etc. 15.](#_Toc521433382)

[18. costs of research 15](#_Toc521433383)

[18.1 Research funding and conflicts of interest 15](#_Toc521433384)

[18.2 Cost sharing for subjects 15](#_Toc521433385)

[18.3. health response and compensation 15](#_Toc521433386)

[19. duration of the study and termination/early discontinuation of the study 15](#_Toc521433387)

[19.1 Study period 15](#_Toc521433388)

[19.2. end of study 16](#_Toc521433389)

[19.3 Early discontinuation of research 16](#_Toc521433390)

[20. storage and use of medical devices and their storage periods 16](#_Toc521433391)

[21. record keeping 16](#_Toc521433392)

[22. publication of research and attribution of results 16](#_Toc521433393)

[22.1 Registering your research plan 16](#_Toc521433394)

[22.2 Attribution of results 17](#_Toc521433395)

[23. research implementation structure 17.](#_Toc521433396)

[24. reference materials and bibliography 17](#_Toc521433397)

[25. appendix 18](#_Toc521433398)

# 0. overview

## Shema

　The groups will be divided according to eligibility criteria and other factors based on the following two shema.

Main eligibility criteria: students of our University's Department of Health Sciences

Registration and allocation

Target number of registered participants: 60

　　Enrolment period: from the date of Ethics Committee approval to 31 January 2024

Group M (20 participants, massage)

(Intervention by exercise stimulus)

Effectiveness assessment: measuring each indicator before and immediately after the intervention

Group C (20 participants, control)

Group P (20 participants, physical stimulation)

## Purpose and significance

　The aim of this study was to examine whether exercise and physical stimulation interventions on the tissues around the knee joint can change the flexibility and gliding properties of the tissues in healthy subjects, using ultrasound imaging equipment.

　If the usefulness of the intervention is confirmed through this study, it will provide a basis for the development of new medical technology and contribute to the development of rehabilitation medicine.

## Target

Students of our University's Faculty of Medicine

## Target number of people to be enrolled and duration of study

　Target number of patients enrolled: 60 (20 in group M, 20 in group P and 20 in group C)

　Enrolment period: from the date of Ethics Committee approval to 31 January 2024, depending on the subject.

　　Duration of the study: from the date of approval by the Ethics Committee to 31 March 2024

## Research design

　□Study design: randomised controlled trial in a parallel design

　□Type of control: no intervention

　□Randomisation: stratified block randomisation method

　□Level of blinding: unblinded

## Assessment items

　　Main endpoints: ultrasound imaging (flexibility of the fat body under the patella, gliding of the medial patellar girdle)

　　Secondary endpoint: other ultrasound imaging data

## Contact us

[Enquiries about test content and registration

Research secretariat: Takashi Kitagawa, Assistant Professor, Department of Physical Therapy, Shinshu University School of Medicine and Health Sciences

　　Post 390-8621

　3-1-1 Asahi, Matsumoto City, Nagano Prefecture

　Tel: 0263-37-2413

# 1. purpose and significance

There have been reports on subjective methods of assessing the gliding properties of periprosthetic tissues in the knee joint using ultrasound imaging, but no objective methods have been devised to date.

The purpose of this study was to quantitatively examine whether exercise and physical stimulation of the tissues surrounding the knee joint can change the flexibility and gliding ability of the tissues using ultrasound imaging in healthy subjects. Specifically, we will target the knee joint, which is prone to contracture (restriction of joint movement) in many diseases, and quantitatively evaluate (1) the flexibility of the infrapatellar fat pad and (2) the gliding of the medial patellar band due to contraction of the vastus medialis muscle, and examine the changes before and after intervention.

　If the usefulness of the intervention is confirmed through this study, it will provide a basis for the development of new medical technology and contribute to the development of rehabilitation medicine.

# 2. background and rationale

　Skeletal muscles and their tendons are the main tissues responsible for the movement of joints in the human body and are one of the many soft tissues that span joints throughout the body. The skeletal muscles and their tendons are one of the many soft tissues that span the joints of the body. While the normal elasticity, flexibility and gliding properties of the skeletal muscles and their tendons do not interfere with joint movement, prolonged periods of rest and bed rest associated with hospitalization and treatment for diseases and other conditions often result in a decline in their function and contractures. In particular, in elderly patients, the proportion of which is increasing significantly in Japan, the early release from bed, smooth return to home and quality of life are adversely affected. Therefore, it is desirable to take appropriate measures to prevent and treat contractures from the early stages of treatment, but the detailed pathogenesis remains unclear and there is no evidence-based treatment or therapy. Although it has been reported that the cause of contractures is largely due to a decrease in muscle-tendon elasticity, flexibility and gliding, previous research by the applicant suggests that degeneration of the fatty tissue surrounding the knee joint also contributes to contractures and dysfunction (Kitagawa T et al, J Med Ultrason 2019, etc.). However, based on the findings of other relevant previous studies, the detailed pathogenesis of contractures remains unclear.

In Japan, where the population is aging, it is one of the most urgent issues from the perspective of extending healthy life expectancy and controlling soaring medical costs to return patients whose physical functions deteriorate due to hospitalization for diseases to society in good health as much as possible (Ministry of Health, Labour and Welfare, Japan, 2018). We have analyzed studies on the early discharge of hospitalized patients, their return to home, and the improvement of their quality of life from various perspectives. We have also found that ultrasound findings of knee joint components are associated with various physical functions (Kitagawa T at al., in press). The aim of this study is to examine the usefulness of physiotherapeutic approaches to the flexibility and gliding of the anterior and medial knee joint surrounding tissues, which tend to cause pain in clinically limited range of motion of the knee joint.

# 3. overview of the test drug

　This is a drug-free study. The aim of this study is to compare the changes in the dynamics of the tissues around the knee joint using ultrasound imaging, before and after intervention, in order to develop a new physiotherapeutic treatment.

# 4. eligibility criteria

Eligible students of the University's Faculty of Medicine who meet all of the following selection criteria and none of the exclusion criteria are eligible for enrolment.

## 4.1 Selection criteria

　Minors are also included because it is necessary to include them in order to ensure the sample size necessary to carry out this study.

A student of the University

　A person who is 18 years of age or older at the time the consent form is obtained.

　(3) Those who have given written consent based on the subject's own free will after receiving sufficient explanation for participation in this study.

　We will recruit students by posting notices on campus noticeboards and by other means.

## 4.2 Exclusion criteria

　　(i) Those with sensory impairment in the lower limbs

　　(2) Patients with a history of neurological or orthopaedic disease of the spine or lower limbs

　　(3) Any other person who is judged by the principal investigator to be unsuitable as a human subject.

　Those with limited knee extension or hypermobility for any reason.

## 4.3 Subjects for whom consent by a substitute is required and the reasons for this

　Considering the age structure of the subjects of this study, minors are also included because it would be difficult to carry out this study without including them.

　Consent shall be obtained from a surrogate when conducting research on the above subjects. The substitute will be selected from those who are considered to be able to represent the subject's wishes and interests, taking into account the subject's family structure. Persons who are considered appropriate as surrogates are listed below.

Parent of the subject

Spouse of the subject

Adult child

Adult siblings or grandchildren

Grandparents

Adult relatives or persons considered to be equivalent to these relatives

# 5. research methods

## 5.1 Research design

　□Study design: randomised controlled trial in a parallel design

　□Type of control: no intervention

　□Randomisation: stratified block randomisation method

　□Level of blinding: unblinded

## 5.2 Outline of the study

Massage and exercise group (group M), physical stimulation group (group P)

Register

Eligibility check

Obtaining consent

Intervention period

Pre-observation period

Tracking period

One single intervention

None in particular

2 weeks

End of experiment

End of intervention

Start of intervention

Control group (Group C)

The pre-observation period, intervention period and follow-up period all follow the same process as above.

No intervention, but a total of two ultrasound imaging sessions with a 10-minute pause for comparison with the intervention group.

## 5.3 How interventions are delivered

Exercise stimulus group; 1/3 of participants

　A massage/manual therapy, quantified in a preliminary experiment, will be performed on the infrapatellar fat pad for approximately 10 minutes. Specifically, a pressure sensor will be used to measure the finger pressure during the manual approach of the interventionist to ensure that a constant pressure is applied.


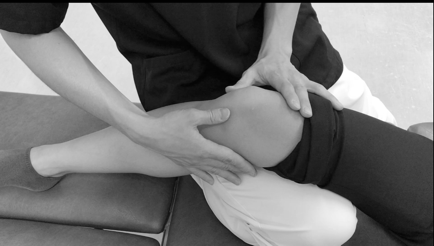
　　　　　
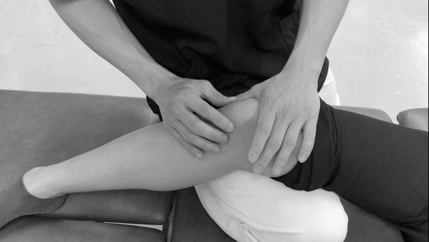


Physical stimulus group; 1/3 of participants

　The infrapatellar fat pad body is subjected to vibration stimulation quantified in the preliminary experiment for approximately 10 minutes. The frequency of the vibration stimulation is quantified by using a device that can quantify the frequency of the vibration stimulation, so that the vibration stimulation is applied at a constant frequency.

Equipment to be used: Slieve MD01 handy massager.

The interventions in the above two groups will be carried out by a researcher (Yuma Aoki) who has been carefully trained beforehand.

Control group; 1/3 of participants

　No specific interventions will be made, but there will be a 10-minute waiting period.

## 5.4 Provisions for concomitant medicines (combination therapy)

Not applicable.

## 5.5 What to do with subjects after completion of the study

　After the completion of this study, the principal investigator will provide the subjects with a summary of the information, including the results obtained in this study, which may be of benefit to medical education.

# 6. method of registration and allocation of subjects

##

## 6.1 Registration of subjects

　The principal investigator or sub-investigator (hereafter referred to as the "principal investigator") will complete the Subject Registration Form and submit it to the Data Management Officer. The data management officer will review the eligibility and notify the researcher in writing of the result of the decision. The researcher registers the subject, attaching the result of the Data Management Officer's decision to the Subject Registration Form.

## 6.2 Allocation method and allocation adjustment factors

　If, in a preliminary experiment, factors are identified that clearly influence the outcome (e.g. gender), then stratified block randomisation by these factors will be used. No pre-allocation adjustment factors will be assumed, as previous studies have shown that absence of a clear history of acute trauma or knee disease is unlikely to affect outcome.

7. endpoints

## 7.1 Primary endpoints

　　Main endpoints: flexibility of the fat body under the patella calculated using ultrasound images and gliding of the medial patellar strut with contraction of the vastus medialis

## 7.2 Secondary endpoints

　Secondary endpoint: other ultrasound findings

The ultrasound imaging described above will be performed by a blinded researcher (Natsumi Ozaki) who will not be informed which intervention the subject has received.

# 8. observations and tests

Observation, examination and reporting schedule (Group M, Group P, Group C)

| Occasion  Inspection, observation and investigation items | Pre | Before | After |
| --- | --- | --- | --- |
|  |  |  |  |
| Obtaining consent | ● |  |  |
| Subject background (age, medical history) | ● |  |  |
| Physical examination (height and weight) | ● |  |  |
| Ultrasound images |  | ● | ● |
| Adverse events | ←△→ | | |

# 9. handling of adverse events

As this is a non-invasive interventional study, no adverse events are expected.

# 10. target number of people to be registered

　Target number of patients enrolled: 60 (20 in group M, 20 in group P and 20 in group C)

　　Enrolment period: from the date of Ethics Committee approval to 31 January 2024, depending on the subject.

　Duration of the study:from the date of approval by the Ethics Committee to 31 March 2024

# 11. statistical matters

## 11.1 Basis for setting the target number of people to be registered

To examine the changes before and after the intervention between the three different groups. The rationale for the number of subjects in each group will be based on sample size calculations. Specifically, a two-way analysis of variance will be used for statistical analysis. The number of levels is 1 (before or after the intervention) and the number of groups is 3. Assuming an effect size of 0.4, a significance level of less than 5% and a power of 0.8, the sample size for each group will be calculated to be 52 participants in total. Assuming that there will be a certain number of subjects who declare that they cannot consent to randomisation and who meet the exclusion criteria, we aim to enroll 60 subjects.

## 11.2 Statistical analysis methods

Analyses of the effectiveness of interventions will be carried out in the Full Analysis Set (FAS).

FAS

The FAS is the analytic population consisting of subjects who do not meet any of the following criteria

a) If you are unable to complete the intervention for any reason

b) If ultrasound imaging was not available before or after the intervention

## 11.3 Analysis items and methods

　A summary of the statistical analysis is given below. The significance level of the test is set at two-sided 0.05. For details, Takashi Kitagawa, who is responsible for the statistical analysis, will prepare the first version of the statistical analysis plan before the first patient is enrolled (first patient in), and if necessary, the second version will be fixed before the data are fixed. If a second version is prepared, it will include a history of changes from the first version.

### 11.3.1 Overview of the analysis

1) Composition of the population for analysis

　 The number of enrolled, eligible, treatment-started and analysed subjects will be calculated by intervention group. Subjects who were unable to complete the intervention and those who were found to be ineligible after enrolment will be counted by reason.

2) Subject background factors and baseline data

　 Subject background factors and baseline data such as age, sex and previous medical history will be ascertained and ultrasound imaging will be performed.

### 11.3.2 Hypothesis-testing analysis of primary endpoints

To determine and compare the effects on the tissues surrounding the knee joint before and after the intervention, we will compare the average of each index between the three groups.

If the p-value of this test is less than 0.05 and the mean value of each indicator after the intervention is higher in group M or P than in group C, we judge that the intervention may be useful.

### 11.3.3 Analysis of secondary endpoints

There is a possibility of conducting a backward-looking secondary outcome analysis of the ultrasound images obtained to inform future research activities.

### 11.3.4 Hypothesis exploratory analysis for primary and secondary endpoints

Not applicable.

# 12. completion and submission of the case report form

Not applicable.

# 13. monitoring

Not applicable.

#

# 14. audit

Not applicable.

# 15. ethical matters

## 15.1 Rules to be observed

　All parties involved in this research will comply with the World Medical Association Declaration of Helsinki and the Ethical Guidelines for Medical Research Involving Human Subjects.

## 15.2 Informed consent

　The researcher will provide the subject (including a substitute if one is required; the same applies below) with a consent explanation document approved by the University's Ethics Committee, provide sufficient written and oral explanations, and obtain the subject's free and voluntary consent in writing.

When information that may affect the consent of subjects is obtained, or when changes are made to the implementation plan, etc. that may affect the consent of subjects, the researcher will promptly provide information to the subjects, confirm in advance the subjects' intentions regarding whether or not to participate in the research, and obtain prior approval from the University's Ethics Committee to revise the consent In addition, we will obtain the prior approval of the University's Ethics Committee, revise the consent explanatory document, etc., and obtain the subject's consent again.

The consent document should include the following information

① The name of the research The name of the research and the fact that the research has been authorized by the head of the research institution

② Name of the research organisation and the name of the principal investigator(s). The name of the research institution and the name of the principal investigator (including the name of the joint research institution and the name of the principal investigator of the joint research institution if the research is to be conducted jointly with another research institution)

③ Purpose and significance of the study

④ The method of the research (including the purpose of use of the samples and information obtained from the research subjects) and duration

⑤ Reasons for being selected as a research subject

⑥ Burden and anticipated risks and benefits to research subjects

⑦ The fact that the research subject may withdraw his/her consent at any time even if he/she has consented to the conduct or continuation of the research (if there are cases where it will be difficult to take measures in accordance with the content of the withdrawal from the research subject, etc., a statement to that effect and the reasons therefor)

⑧ A statement that the research subject will not be treated adversely by not consenting to the conduct or continuation of the research or by withdrawing consent.

⑨ How we disclose information about our research

⑩ (ix) The fact that research subjects, etc. may, upon request, obtain or inspect materials related to the research protocol and research methods to the extent that such access does not interfere with the protection of the personal information of other research subjects, etc. and the originality of the research, and the method of obtaining or inspecting such materials.

⑪ Handling of personal information, etc. (including the method of anonymisation, if any)

⑫ Methods of storage and disposal of samples and information

⑬ Status of conflicts of interest relating to the research of researchers and others, including sources of funding for research, conflicts of interest relating to the research of research institutions and earnings of individuals

⑭ Response to consultations from research subjects and related persons

⑮ If there is any financial burden or gratuity to be paid to the research subjects, etc., a statement to that effect and the details thereof

⑯ In the case of research involving medical treatment beyond normal practice, matters relating to other methods of treatment, etc.

⑰ In the case of research involving medical treatment that goes beyond normal medical practice, measures relating to the provision of medical care to research subjects after the research has been conducted

⑱ The results of the research pertaining to the research subject (including incidental findings) if the conduct of the research may lead to important findings concerning the health of the research subject, genetic characteristics that may be passed on to offspring, etc. Treatment of

⑲ In the case of invasive research, the existence and nature of any compensation for damage to health caused by the research

⑳ When there is a possibility that the samples and information obtained from the research subjects will be used for future research that is not specified at the time of receiving consent from the research subjects, etc., or that the information will be provided to other research institutions, that fact and the contents assumed at the time of receiving consent

## 15.3 Protection of personal data

When handling information related to the implementation of the research, a correspondence table will be prepared with numbers unrelated to the personal information of the subjects, and the information will be anonymized to give due consideration to the protection of the confidentiality of the subjects. The correspondence table will be strictly managed by the personal information manager and will not be provided to outside parties. When the results of the research are published, information that can identify the subjects will not be included. In addition, we will not use the information about the subjects obtained in the study for any purpose other than the purpose of the study.

## 15.4 Storage of information, etc.

The ultrasound images and other data relating to this study will be stored under the supervision of the principal investigator and the information (materials) will be stored in a lockable vault in the Department of Physiotherapy, Faculty of Medicine and Health Sciences for 10 years after publication of the results in a paper or other publication. At the end of the storage period, the data will be disposed of in anonymised form. Paper data will be disposed of in anonymised form using a shredder or similar device and electronic data will be completely erased.

# 16. records relating to the provision of samples and information

As no samples or information are exchanged, no records are made.

# 17. changes to the research protocol etc.

　Any changes or revisions to the research protocol or the consent explanatory document for this research must be approved in advance by the University's Ethics Committee.

# 18. costs of research

## 18.1 Research funding and conflicts of interest

This study is funded by the Department of Physical Therapy, to which the principal investigator belongs, and by JSPS Grant-in-Aid for Scientific Research 19K24282. In addition, the researcher in charge of this study shall declare the necessary information to the Shinshu University Conflict of Interest Management Committee for Clinical Research in accordance with the "Procedure for Applying for Ethical Review of Shinshu University School of Medicine", and obtain its review and approval.

## 18.2 Subjects' expenses or gratuities

The equipment used in this study will be provided by the University, so there will be no cost to the subject for participating in the study.

## 18.3 Response to and compensation for health hazards

This is a non-invasive interventional study with no potential for adverse events and will use only the subject's ultrasound images. Therefore, no compensation will be prepared for this study as it is not expected to cause any health problems to the subjects. The approval of the Ethics Committee of the University will be obtained for this matter, and the subjects will be fully informed and asked to participate in this study with their understanding and consent.

# 19. duration of the study and termination/early discontinuation of the study

## 19.1 Research period

　Enrolment period: from the date of Ethics Committee approval to 31 January 2024, depending on the subject.

　　Duration of the study: after passing the ethical review - 31 March 2024

## 19.2 Completion of the study

　The study will be terminated when the data fixation of the last enrolled subject is completed, and the principal investigator will promptly submit a report on the termination of the study to the Medical Director.

## 19.3 Early termination of research

　The Principal Investigator will consider whether or not to continue to conduct the research if any of the following apply

1. If it is judged to be extremely difficult to reach the expected number of subjects due to the difficulty of inclusion
2. the purpose of the research is achieved before the expected number of subjects or the expected duration is reached
3. If the researcher deviates from the ethical guidelines or the research protocol, or breaches a contract, and it is judged difficult to continue the research appropriately.
4. When the University's Ethics Committee instructs changes to the implementation plan, etc., and it is judged to be difficult to accept these changes.

The principal investigator will discontinue the research if the University Ethics Committee recommends or directs that the research be discontinued. When the decision to discontinue the research is made, the researcher will promptly report in writing to the Dean of the Faculty of Medicine with the reasons for the decision.

# 20. storage and use of medical devices and their storage periods

Ultrasound imaging equipment will be used in this study. The ultrasound images and other data relating to this study will be stored under the supervision of the principal investigator and the information (materials) will be stored in a lockable vault in the Department of Physiotherapy, Faculty of Medicine and Health Sciences for 10 years after publication of the results in a paper or other publication. At the end of the storage period, the data will be disposed of in anonymised form. Paper data will be disposed of in anonymised form using a shredder or similar device and electronic data will be completely erased.

# 21. record keeping

The principal investigator will retain important documents related to the implementation of the research, etc. (e.g., copies of application documents, notification documents from the hospital director, copies of various application forms and reports, consent forms, and other documents or records necessary to ensure the reliability of the data) for 10 years after the publication of the results in a paper or other publication. After that, they will be disposed of with care and with respect to personal data.

# 22. publication of research and attribution of results

## 22.1 Registering your research plan

　The study will be registered as a clinical trial in the database of the UMIN Clinical Trials Registration System (http://www.umin.ac.jp/ctr/index-j.htm), which will be updated as appropriate to reflect changes in the study protocol and the progress of the study, and the results of the study will be registered without delay when the study is completed.

## 22.2 Attribution of results

　　The results of this research shall belong to the Faculty of Medicine and Health Sciences, Shinshu University. The principal investigator will publish the results of this study through presentations at relevant conferences.

# 23. research implementation structure

　This research will be carried out under the following structure.

Research supervisor

○Takashi Kitagawa, Assistant Professor, Department of Physical Therapy, School of Health Sciences, Shinshu University

(responsible for data analysis in a blinded environment)

Natsumi Ozaki, Department of Physical Therapy, School of Health Sciences, Shinshu University

(Responsible for ultrasound imaging in a blinded environment)

Yuma Aoki, Department of Physical Therapy, School of Health Sciences, Shinshu University

(Intervention Officer)

Kimi Nishizawa, Associate Professor, Department of Physical Therapy, School of Health Sciences, Shinshu University

(Responsible for general data analysis)

(○ Principal Investigator)

Personal Information Manager

Kazuaki Koyake, Assistant Professor, epartment of Physical Therapy, School of Health Sciences, Shinshu University

Data Management Officer

Kumi Nishizawa, Associate Professor, epartment of Physical Therapy, School of Health Sciences, Shinshu University

# 24. reference materials and bibliography

1) Kitagawa T, et al., Use of ultrasonography to evaluate the dynamics of the infrapatellar fat pad after anterior cruciate ligament reconstruction: a J Med Ultrason (2001) 46(1) 147-151.

2) Mikkilineni H, et al., Ultrasound evaluation of infrapatellar fat pad impingement: an exploratory prospective study. Knee, 25 (2), Knee, 25 (2), 279-285.

3) Kitagawa T, et al., Relationship between the deep flexion of the knee joint and the dynamics of the infrapatellar fat pad after anterior cruciate J Phys Ther Sci. 31(7) 569-572.

4) Mace J et al., Infrapatellar fat pad syndrome: A review of anatomy, function, treatment and dynamics. Acta Orthop Belg, 82 (1), 94-101.

25. appendix

Not applicable.
