# Supplementary material for: Effect of physical therapy on the flexibility of the infrapatellar fat pad: A single-blind randomised controlled trial
Source: PLoS One. 2022 Mar 17;17(3):e0265333. doi: 10.1371/journal.pone.0265333 (PMC8929552; doi:10.1371/journal.pone.0265333)
Supplement: S2 File — (DOCX) [file pone.0265333.s004.docx]

**自主臨床研究**

**超音波画像を用いた膝関節の周辺組織の滑走性評価**

**および治療方法の開発**

**研　究　実　施　計　画　書**

研究責任者 信州大学医学部　保健学科理学療法学専攻

助教　北川孝

| 版数 | 作成日 |
| --- | --- |
| 第1.0版 | 2020年1月30日 |
|  |  |
|  |  |
|  |  |
|  |  |

目次

[0. 概要 5](#_Toc521433322)

[0.1. シェーマ 5](#_Toc521433323)

[0.2. 目的及び意義 5](#_Toc521433324)

[0.3. 対象 5](#_Toc521433325)

[0.4. 目標登録対象者数と試験期間 5](#_Toc521433326)

[0.5. 研究デザイン 6](#_Toc521433327)

[0.6. 評価項目 6](#_Toc521433328)

[0.7. 問い合わせ先 6](#_Toc521433329)

[1. 目的及び意義 7](#_Toc521433330)

[2. 背景と根拠 7](#_Toc521433331)

[3. 試験薬の概要 8](#_Toc521433332)

[4. 適格性の基準 8](#_Toc521433333)

[4.1.　選択基準 8](#_Toc521433334)

[4.2.　除外基準 8](#_Toc521433335)

[4.3.　代諾者による同意が必要な被験者とその理由 8](#_Toc521433335)

[5. 研究の方法 9](#_Toc521433337)

[5.1.　研究デザイン 9](#_Toc521433338)

[5.2.　研究のアウトライン 9](#_Toc521433339)

[5.3.　介入の実施方法 9](#_Toc521433340)

[5.4. 併用薬（併用療法）についての規定 10](#_Toc521433341)

[5.5. 研究終了後の被験者への対応 10](#_Toc521433346)

[6. 対象者登録・割付の方法 10](#_Toc521433347)

[6.1.　対象者登録 10](#_Toc521433348)

[6.2.　割付方法と割付調整因子 10](#_Toc521433349)

[7. 評価項目（エンドポイント） 10](#_Toc521433350)

[7.1.　主要評価項目 10](#_Toc521433351)

[7.2.　副次的評価項目 11](#_Toc521433352)

[8. 観察・検査項目 11](#_Toc521433353)

[9. 有害事象の取り扱い 11](#_Toc521433354)

[10. 目標登録対象者数 11](#_Toc521433361)

[11. 統計的事項 11](#_Toc521433362)

[11.1.　目標登録対象者数の設定根拠 11](#_Toc521433363)

[11.2.　統計解析方法 12](#_Toc521433364)

[11.3.　解析項目・方法 12](#_Toc521433365)

[11.3.1.　解析対象の概要 12](#_Toc521433366)

[11.3.2.　主要評価項目に関する仮説検証的解析 12](#_Toc521433367)

[11.3.3.　副次評価項目に関する解析 12](#_Toc521433368)

[11.3.4.　主要評価項目及び副次評価項目に関する仮説探索的解析 13](#_Toc521433369)

[12. 対象者報告書の記入と提出 13](#_Toc521433370)

[13. モニタリング 13](#_Toc521433373)

[14. 監査 13](#_Toc521433374)

[15. 倫理的事項 13](#_Toc521433375)

[15.1.　遵守すべき諸規則 13](#_Toc521433376)

[15.2.　インフォームド・コンセント 13](#_Toc521433377)

[15.3.　個人情報の保護 14](#_Toc521433378)

[15.4試料･情報の保存等について 15](#_Toc521433379)

[16. 試料・情報の提供に関する記録事項 15](#_Toc521433381)

[17. 研究実施計画書等の変更 15](#_Toc521433382)

[18. 研究の費用 15](#_Toc521433383)

[18.1.　研究資金及び利益相反 15](#_Toc521433384)

[18.2.　被験者の費用負担 15](#_Toc521433385)

[18.3.　健康被害への対応と補償 15](#_Toc521433386)

[19. 研究期間と研究の終了・早期中止 15](#_Toc521433387)

[19.1.　研究期間 15](#_Toc521433388)

[19.2.　研究の終了 16](#_Toc521433389)

[19.3.　研究の早期中止 16](#_Toc521433390)

[20. 医療機器等の保存及び使用方法並びに保存期間 16](#_Toc521433391)

[21. 記録の保存 16](#_Toc521433392)

[22. 研究の公表と成果の帰属 16](#_Toc521433393)

[22.1.　研究計画の登録 16](#_Toc521433394)

[22.2.　成果の帰属 17](#_Toc521433395)

[23. 研究実施体制 17](#_Toc521433396)

[24. 参考資料・参考文献 17](#_Toc521433397)

[25. 付録 18](#_Toc521433398)

# 0. 概要

## シェーマ

　以下の2つのシェーマに基づき、適格基準等に応じて群分けを行う。

主な適格基準：本学保健学科学生

登録・割付け

目標登録対象者数：60名

　　対象者登録期間：倫理委員会承認日より2024年1月31日

M群（20名、

運動刺激による介入）

効果判定：介入開始前、介入直後に各指標を計測する

C群（20名、コントロール）

P群（20名、

物理刺激による介入）

## 目的及び意義

　本研究の目的は、健常者を対象として、膝関節周辺組織に対する運動・物理刺激介入によって組織の柔軟性・滑走性が変化するかどうかを、超音波画像診断装置を用いて検証することである。

　本研究を通じ、介入の有用性が確認されれば新しい医療技術開発の基礎となり、リハビリテーション医学の発展に寄与するものと思われる。

## 対象

本学医学部の学生

## 目標登録対象者数と試験期間

　目標登録対象者数：60名（M群20名、P群20名、C群20名）

　対象者登録期間：対象者に応じ倫理委員会承認日より2024年1月31日

　試験実施期間：倫理委員会承認日より2024年3月31日

## 研究デザイン

　□研究デザイン：並行デザインでのランダム化比較試験

　□対照の種類：介入なし

　□ランダム化：層別ブロックランダム化法

　□盲検化のレベル：非盲検

## 評価項目

　主要評価項目：超音波画像(膝蓋下脂肪体の柔軟性、内側膝蓋支帯の滑走性)

　副次評価項目：その他超音波画像データ

## 問い合わせ先

【試験内容、登録に関する問い合わせ】

研究事務局：信州大学医学部保健学科　理学療法学専攻　助教　北川孝

　〒390-8621

　長野県松本市旭3-1-1

　TEL：0263-37-2413

# 1. 目的及び意義

膝関節における超音波画像を用いた関節周辺組織の滑走性評価手法については主観的なものに関する報告はあるが、客観的な手法についてはこれまで考案されていない。

本研究の目的は、健常者を対象として、膝関節周辺組織に対する運動・物理刺激介入によって組織の柔軟性・滑走性が変化するかどうかを、超音波画像を用いて定量的に検証することである。具体的には多くの疾患で拘縮(関節の動きの制限)が生じやすい膝関節をターゲットとし、①膝蓋下脂肪体の柔軟性、ならびに②内側広筋の収縮に伴う内側膝蓋支帯の滑走性の2つを定量的に評価し、介入前後の変化を検討する。

　本研究を通じ、介入の有用性が確認されれば新しい医療技術開発の基礎となり、リハビリテーション医学の発展に寄与するものと思われる。

# 2. 背景と根拠


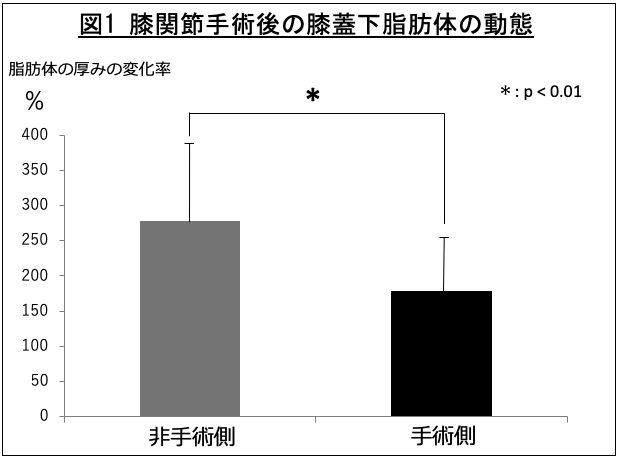
　骨格筋およびその腱は人体の関節の運動を行うために主要な組織であり、全身の関節をまたぐように数多く存在する軟部組織の1つである。正常な伸張性・柔軟性および近隣組織間との滑走性が保たれているうちは関節の運動に支障をきたさないが、疾患などにより入院・治療に伴う安静・臥床期間が長期化するとそれらの機能が低下し、拘縮を呈することがしばしばみられ、特に本邦における人口比率が著しく増加している高齢者においては早期離床・円滑な在宅復帰・生活の質に対し悪影響を及ぼす。そのため治療早期から拘縮予防・治療のために適切な対処が望まれるが、その詳細な病態は不明瞭なままであり、エビデンスの高い治療・処置は存在していない。この拘縮の原因は筋腱の伸張性・柔軟性あるいは滑走性の低下が大きく寄与していると報告されているが、申請者の先行研究では、膝関節において、関節周辺にある脂肪組織の変性もまた拘縮や機能障害に影響を及ぼしていることが示唆された(Kitagawa Tら、J Med Ultrason 2019など) (図1)。しかし他の関連する先行研究の知見を踏まえても、拘縮の詳細な病態は未だ不明瞭なままである。

社会の高齢化が進む本邦において、疾患などによる入院のために身体機能が低下する患者を少しでも良い健康状態で社会へ復帰させることは健康寿命の延伸・高騰する医療費抑制といった観点からも喫緊の課題の1つである(厚生労働省, 2018)。これまで本研究責任者は、入院患者の早期退院や在宅復帰、その後の生活の質の向上に関する研究を多様な視点から解析してきた。また膝関節構成体の超音波画像所見が様々な身体機能と関連することを突き止めてきた(Kitagawa T at al. 投稿中)。この度、臨床的に膝関節の可動域制限において痛みを生じやすい膝関節前面および内側の周辺組織の柔軟性・滑走性に対する理学療法的アプローチの有用性を検証することを図るものである。

# 3. 試験薬の概要

　本研究は薬剤の使用を伴わない研究である。本研究は新しい理学療法的治療法を開発すべく、超音波画像を用いた膝関節周辺組織の動態の変化を介入前後あるいは有無にて比較・検討するものである。

# 4. 適格性の基準

本学医学部の学生で以下の選択基準をすべて満たし、さらに除外基準のいずれにも該当しない対象者を登録適格例とする。

## 4.1.　選択基準

　本研究の遂行に必要なサンプルサイズを担保するためには、未成年者を対象に加える必要性もあるため、未成年者も対象としている。

①本学の学生である者

　②同意書取得時において年齢が18歳以上の者

　③本研究への参加にあたり十分な説明を受けた後、被験者本人の自由意思に基づき文書による同意が得られた者

　・対象者のリクルートは学内掲示板への案内告知等の手段にて行っていく。

## 4.2.　除外基準

　①下肢に感覚障害を有する者

　②脊椎・下肢の神経学的・整形学的疾患の既往がある者

　③その他、研究責任者が被験者として不適当と判断した者

　④何らかの理由により膝関節伸展制限あるいは過可動性がある者

## 4.3代諾者による同意が必要な被験者とその理由

　本研究の対象の年齢構成を考慮すると、未成年者を対象に加えなければ本研究の遂行が困難であるため、未成年者も対象としている。

　なお、上記被験者を対象として研究を実施する際には、代諾者からの同意を得るものとする。代諾者は、被験者の家族構成等を勘案して、被験者の意思及び利益を代弁できると考えられる者から選択する。代諾者として適切と考えられる者を以下に示す。

被験者の親

被験者の配偶者

成人の子

成人の兄弟姉妹もしくは孫

祖父母

成人の親族又はそれらの親近者に準ずると考えられる者

# 5. 研究の方法

## 5.1.　研究デザイン

　□研究デザイン：並行デザインでのランダム化比較試験

　□対照の種類：介入なし

　□ランダム化：層別ブロックランダム化法

　□盲検化のレベル：非盲検

## 5.2.　研究のアウトライン

マッサージ・運動刺激群(M群)、物理刺激群(P群)

介入終了

介入開始

登録

適格性確認

同意取得

介入期間

前観察期間

追跡期間

1回単発の介入

特になし

2週間

実験終了

コントロール群(C群)

前観察期間・介入期間・追跡期間、全て上記と同様の流れである。

介入は行わないが、介入群との比較のため、10分間の間をはさみ超音波画像撮影を計2回行う。

## 5.3.　介入の実施方法

【運動刺激群; 参加者の1/3】

　膝蓋下脂肪体に対し約10分間、予備実験で定量化したマッサージ・徒手療法を行う。具体的には、圧センサーを用いて介入者の徒手アプローチ時の指の圧力を計測し、一定の圧を加えるようにする。


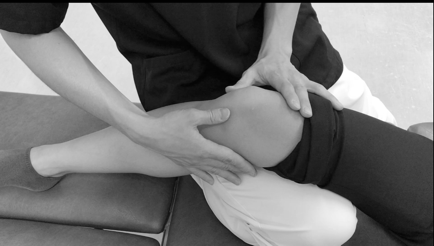
　　　　　
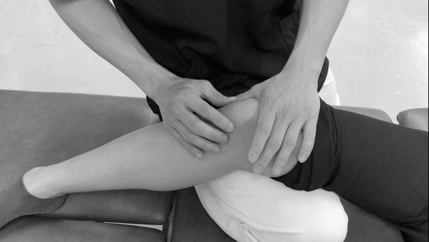


【物理刺激群; 参加者の1/3】

　膝蓋下脂肪体に対し約10分間、予備実験で定量化した振動刺激を行う。具体的には、振動刺激の周波数を定量化できる機器を用い、一定の周波数での振動刺激を加えるようにする。

使用予定機器：スライヴ ハンディマッサージャー MD01

上記2群への介入は、事前に入念を行った研究担当者(青木優真)が担当する。

【コントロール群; 参加者の1/3】

　特に介入は行わないが、10分間の待機時間をとる。

## 5.4. 併用薬（併用療法）についての規定

該当なし。

## 5.5. 研究終了後の被験者への対応

　本研究終了後は、この研究で得られた成果も含めて、研究責任者は被験者に対し医学教育に有益であると思われる情報を要約して提供する。

# 6. 対象者登録・割付の方法

##

## 6.1.　対象者登録

　研究責任者又は研究分担者（以下、研究担当者）は、対象者登録票に必要事項を記入し、データマネジメント担当者へ提出する。データマネジメント担当者は適格性の審査を行い、判定結果を文書で研究担当者に通知する。研究担当者は、対象者登録票にデータマネジメント担当者の判定結果を添付し、対象者を登録する。

## 6.2.　割付方法と割付調整因子

　予備実験にて、明らかにアウトカムに影響を及ぼすと思われる因子(例：性別など)が分かれば、その因子による層別ブロックランダム化を行う。先行研究では明らかな急性外傷や膝関節疾患の既往がなければアウトカムには影響がないと思われるため、事前に割付調整因子は想定していない。

7. 評価項目（エンドポイント）

## 7.1.　主要評価項目

　主要評価項目：超音波画像を用いて算出される膝蓋下脂肪体の柔軟性および内側広筋の収縮に伴う内側膝蓋支帯の滑走性

## 7.2.　副次的評価項目

　副次評価項目：その他超音波画像所見

上述した超音波画像の撮影は、対象者がどちらの介入を受けたか知らされていない、盲検化された研究担当者(尾﨑夏実)が行う。

# 8. 観察・検査項目

観察・検査・報告スケジュール(M群, P群, C群)

| 時期  検査・観察・調査項目 | 前観察期間 | 介入前 | 介入後 |  |
| --- | --- | --- | --- | --- |
|  |  |  |  |  |
| 同意取得 | ● |  |  |  |
| 被験者背景(年齢、既往歴) | ● |  |  |  |
| 身体所見(身長、体重) | ● |  |  |  |
| 超音波画像 |  | ● | ● |  |
| 有害事象 | ←△→ | | | |

# 9. 有害事象の取り扱い

本研究では有害事象になりえる程度の侵襲を伴わない介入研究であるため、有害事象は想定されない。

# 10. 目標登録対象者数

　目標登録対象者数：60名（M群20名、P群20名、C群20名）

　対象者登録期間：対象者に応じ倫理委員会承認日より2024年1月31日

　試験実施期間：倫理委員会承認日より2024年3月31日

# 11. 統計的事項

## 11.1.　目標登録対象者数の設定根拠

異なる3群間の介入前後での変化の検討を行う。各群の対象者数の根拠はサンプルサイズの計算に基づく。具体的には、統計学的解析に二元配置分散分析を用いる予定である。水準数は1(介入前or後)であり、群数は3である。効果量を0.4、有意水準を5%未満、検出力を0.8とすると各群のサンプルサイズは合計52名必要と算出される。ランダム化の同意を得られないと表明する対象者、除外基準に該当する者がある程度含まれることを想定し、60名の登録を目標とした。

## 11.2.　統計解析方法

介入の有効性の解析は最大解析対象集団（FAS: Full Analysis Set）を対象として行う。

FAS

下記事項のいずれかに該当する被験者を除いて構成される解析対象集団をFASとする。

a)　　何らかの理由で介入を完了できなかった場合

b)　　介入前後で超音波画像の撮影ができなかった場合

## 11.3.　解析項目・方法

　以下に統計解析の概要を示す。検定の有意水準は両側0.05とする。詳細については統計解析責任者である北川孝が第一対象者の登録（First patient in）までに統計解析計画書第1版を作成し、必要に応じてデータ固定までにその第2版を固定する。第2版を作成する場合には第1版からの変更履歴を付す。

### 11.3.1.　解析対象の概要

1）解析対象集団の構成

　 登録対象者数、適格対象者数、治療開始対象者数、解析対象者数を介入群別に算出する。介入を完了できなかった対象者及び登録後に不適格が判明した対象者については、理由別に集計する。

2）被験者背景因子及びベースラインデータ

　 被験者背景因子及びベースラインデータとして、年齢・性別・既往歴を把握するとともに、超音波画像撮影を行う。

### 11.3.2.　主要評価項目に関する仮説検証的解析

介入前後の膝関節周辺組織への影響の判定・比較を行うために3群間における各指標の平均の比較を行う。

この検定のｐ値が0.05よりも小さく、C群よりもM群またはP群の介入後の各指標の平均値が高いときに、介入が有用である可能性があると判断する。

### 11.3.3.　副次評価項目に関する解析

今後の研究活動に活かすべく、得られた超音波画像の後方視的に副次的アウトカムとして解析を行う可能性がある。

### 11.3.4.　主要評価項目及び副次評価項目に関する仮説探索的解析

該当なし。

# 12. 症例報告書の記入と提出

該当なし。

# 13. モニタリング

該当なし。

#

# 14. 監査

該当なし。

# 15. 倫理的事項

## 15.1.　遵守すべき諸規則

　本研究の関係者は「世界医師会ヘルシンキ宣言」および「人を対象とする医学系研究に関する倫理指針」を遵守する。

## 15.2.　インフォームド・コンセント

　研究担当者は、本学倫理委員会で承認の得られた同意説明文書を被験者（代諾者が必要な場合は代諾者を含む、以下同じ）に渡し、文書および口頭による十分な説明を行い、被験者の自由意思による同意を文書で取得する。

研究担当者は、被験者の同意に影響を及ぼす情報が得られたときや、被験者の同意に影響を及ぼすような実施計画等の変更が行われるときは、速やかに被験者に情報提供し、研究に参加するか否かについて被験者の意思を予め確認するとともに、事前に本学倫理委員会の承認を得て同意説明文書等の改訂を行い、被験者の再同意を得ることとする。

なお、同意説明文書は、以下の内容を含むものとする。

① 研究の名称及び当該研究の実施について研究機関の長の許可を受けている旨

② 研究機関の名称及び研究責任者の氏名（他の研究機関と共同して研究を実施する場合には、共同研究機関の名称及び共同研究機関の研究責任者の氏名を含む。）

③ 研究の目的及び意義

④ 研究の方法（研究対象者から取得された試料・情報の利用目的を含む。）及び期間

⑤ 研究対象者として選定された理由

⑥ 研究対象者に生じる負担並びに予測されるリスク及び利益

⑦ 研究が実施又は継続されることに同意した場合であっても随時これを撤回できる旨（研究対象者等からの撤回の内容に従った措置を講じることが困難となる場合があるときは、その旨及びその理由）

⑧ 研究が実施又は継続されることに同意しないこと又は同意を撤回することによって　研究対象者等が不利益な取扱いを受けない旨

⑨ 研究に関する情報公開の方法

⑩ 研究対象者等の求めに応じて、他の研究対象者等の個人情報等の保護及び当該研究の独創性の確保に支障がない範囲内で研究計画書及び研究の方法に関する資料を入　手又は閲覧できる旨並びにその入手又は閲覧の方法

⑪ 個人情報等の取扱い（匿名化する場合にはその方法を含む。）

⑫ 試料・情報の保管及び廃棄の方法

⑬ 研究の資金源等、研究機関の研究に係る利益相反及び個人の収益等、研究者等の研究に係る利益相反に関する状況

⑭ 研究対象者等及びその関係者からの相談等への対応

⑮ 研究対象者等に経済的負担又は謝礼がある場合には、その旨及びその内容

⑯ 通常の診療を超える医療行為を伴う研究の場合には、他の治療方法等に関する事項

⑰ 通常の診療を超える医療行為を伴う研究の場合には、研究対象者への研究実施後における医療の提供に関する対応

⑱ 研究の実施に伴い、研究対象者の健康、子孫に受け継がれ得る遺伝的特徴等に関する重要な知見が得られる可能性がある場合には、研究対象者に係る研究結果（偶発的所見を含む。）の取扱い

⑲ 侵襲を伴う研究の場合には、当該研究によって生じた健康被害に対する補償の有無　及びその内容

⑳ 研究対象者から取得された試料・情報について、研究対象者等から同意を受ける時点では特定されない将来の研究のために用いられる可能性又は他の研究機関に提供する可能性がある場合には、その旨と同意を受ける時点において想定される内容

## 15.3.　個人情報の保護

研究実施に係る情報を取扱う際は、被験者の個人情報とは無関係の番号を付して、対応表を作成し、匿名化を行い被験者の秘密保護に十分配慮する。対応表は個人情報管理者が厳重に管理し、外部への提供は行わない。研究の結果を公表する際は、被験者を特定できる情報を含まないようにする。また、研究の目的以外に、研究で得られた被験者の情報を使用しない。

## 15.4情報の保存等について

本研究に係る超音波画像等のデータは、研究責任者のもと、情報（資料）は論文等の成果発表後10年間、医学部保健学科理学療法学専攻の施錠可能な保管庫に保管する。保管期間終了後、データは、匿名化されたまま処分する。紙データは匿名化されたままシュレッダー等を用いて廃棄し、電子データはデータを完全に消去する。

# 16. 試料・情報の提供に関する記録事項

試料・情報の授受を行わない為、記録の作成は行わない。

# 17. 研究実施計画書等の変更

　本研究の研究実施計画書や同意説明文書の変更または改訂を行う場合は、あらかじめ本学倫理委員会の承認を得る。

# 18. 研究の費用

## 18.1.　研究資金及び利益相反

本研究は、研究責任者が所属する理学療法学専攻の教育研究費およびJSPS科研費 19K24282の助成を受けて実施する。また、本研究の研究担当者は、「信州大学医学部倫理審査申請の手順」にしたがって、信州大学臨床研究に係る利益相反マネジメント委員会に必要事項を申告し、その審査と承認を得るものとする。

## 18.2.　被験者の費用負担又は謝礼

本研究で用いる機器は学内で準備されるため、研究に参加することによる対象者の費用負担は発生しない。

## 18.3.　健康被害への対応と補償

本研究は有害事象を生じ得るような侵襲を伴わない介入研究であり、被験者の超音波画像のみを利用するものである。従って、本研究に伴う被験者への健康被害は発生しないと考えられるため、補償は準備しない。本件について本学倫理委員会の承認を得るとともに、被験者に十分説明し、理解と同意の上で本研究への参加を求めることとする。

# 19. 研究期間と研究の終了・早期中止

## 19.1.　研究期間

　対象者登録期間：対象者に応じ倫理委員会承認日より2024年1月31日

　研究実施期間：倫理審査通過後～2024年3月31日

## 19.2.　研究の終了

　最終登録被験者のデータ固定が終了した時点で本研究の終了とし、研究責任者は速やかに研究終了報告書を医学部長に提出する。

## 19.3.　研究の早期中止

　研究担当者は、以下の事項に該当する場合は、研究実施継続の可否を検討する。

1. 被験者の組み入れが困難で、予定対象者数に達することが極めて困難であると判断されたとき
2. 予定対象者数または予定期間に達する前に、研究の目的が達成されたとき
3. 倫理指針及び研究実施計画書からの逸脱、契約違反等を行い、適切な研究継続が困難と判断された場合
4. 本学倫理委員会により、実施計画等の変更の指示があり、これを受入れることが困難と判断されたとき

研究責任者は、本学倫理委員会により中止の勧告あるいは指示があった場合は、研究を中止する。また、研究の中止を決定した時は、速やかに医学部長にその理由とともに文書で報告する。

# 20. 医療機器等の保存及び使用方法並びに保存期間

本研究では超音波画像診断装置を使用する。本研究に係る超音波画像等のデータは、研究責任者のもと、情報（資料）は論文等の成果発表後10年間、医学部保健学科理学療法学専攻の施錠可能な保管庫に保管する。保管期間終了後、データは、匿名化されたまま処分する。紙データは匿名化されたままシュレッダー等を用いて廃棄し、電子データはデータを完全に消去する。

# 21. 記録の保存

研究責任者は、研究等の実施に係わる重要な文書（申請書類の控え、病院長からの通知文書、各種申請書・報告書の控、同意書、その他データの信頼性を保証するのに必要な書類または記録等）を、論文等の成果発表後10年間保存する。その後は個人情報に注意して廃棄する。

# 22. 研究の公表と成果の帰属

## 22.1.　研究計画の登録

　本研究は、UMIN 臨床試験登録システム（http://www.umin.ac.jp/ctr/index-j.htm）のデータベースへ臨床試験登録し、研究実施計画の変更および研究の進捗に応じて適宜更新し、研究を修了したときは遅滞なく当該試験の結果を登録する。

## 22.2.　成果の帰属

　本研究の成果は、信州大学医学部保健学科に帰属するものとする。研究責任者は、本研究の成果を関連学会での発表を通じて公表する。

# 23. 研究実施体制

　本研究は、以下の体制で実施する。

【研究担当者】

○　北川　孝　　　　信州大学医学部医学部保健学科理学療法学専攻・助教

（盲検化でのデータ解析担当）

尾﨑　夏実　　　信州大学医学部医学部保健学科理学療法学専攻・学生

（盲検化での超音波画像撮影担当）

青木　優真　　　信州大学医学部医学部保健学科理学療法学専攻・学生

（介入担当）

西澤　公美　　　信州大学医学部医学部保健学科理学療法学専攻・准教授

（データ全般の解析担当）

（○ 研究責任者）

【個人情報管理者】

小宅　一彰　　　信州大学医学部医学部保健学科理学療法学専攻・助教

【データマネジメント担当者】

西澤　公美　　　信州大学医学部医学部保健学科理学療法学専攻・准教授

# 24. 参考資料・参考文献

1) Kitagawa T, et al., Use of ultrasonography to evaluate the dynamics of the infrapatellar fat pad after anterior cruciate ligament reconstruction: a feasibility study. J Med Ultrason (2001) 46(1) 147-151.

2) Mikkilineni H, et al., Ultrasound ﻿evaluation of infrapatellar fat pad impingement: an exploratory prospective study. Knee, 25 (2), 279-285.

3) Kitagawa T, et al., Relationship between the deep flexion of the knee joint and the dynamics of the infrapatellar fat pad after anterior cruciate ligament reconstruction via ultrasonography. J Phys Ther Sci. 31(7) 569-572.

4) Mace J et al., Infrapatellar fat pad syndrome: A review of anatomy, function, treatment and dynamics. Acta Orthop Belg, 82 (1), 94-101.

25. 付録

該当なし。
